# Spatial transcriptomics of the epipharynx in long COVID identifies SARS-CoV-2 signalling pathways and the therapeutic potential of epipharyngeal abrasive therapy

**DOI:** 10.1038/s41598-025-92908-7

**Published:** 2025-03-12

**Authors:** Kensuke Nishi, Shohei Yoshimoto, Takayuki Tanaka, Shoichi Kimura, Toshiyuki Tsunoda, Akira Watanabe, Kaori Teranaka, Yo Oguma, Hanako Ogawa, Takumi Kumai, Takafumi Yamano

**Affiliations:** 1 Section of Otolaryngology, Department of Medicine, Fukuoka Dental College, Fukuoka, 814-0193 Japan; 2 Oral Medicine Research Center, Fukuoka Dental College, Fukuoka, 814-0193 Japan; 3 Nishi Otolaryngology Clinic, Fukuoka, 814-0031 Japan; 4 Section of Pathology, Department of Morphological Biology, Division of Biomedical Sciences, Fukuoka Dental College, Fukuoka, 814-0193 Japan; 5 Department of Otolaryngology, Faculty of Medicine, Fukuoka University, Fukuoka, 814-0180 Japan; 6 Department of Cell Biology, Faculty of Medicine, Fukuoka University, Fukuoka, 814-0180 Japan; 7 CyberomiX Inc., Kyoto, 602-8407 Japan; 8 Department of Innovative Head and Neck Cancer Research and Treatment, Asahikawa Medical University, Asahikawa, 078-8510 Japan

**Keywords:** Long COVID-19, Spatial transcriptomics, Visium HD, Chronic epipharyngitis, SARS-CoV-2 signalling pathway, Epipharyngeal abrasive therapy (EAT), Gene expression profiling, Viral infection, Translational research, Infection

## Abstract

In this study, the critical role of the epipharynx in managing long-term coronavirus disease 2019 (COVID-19), and in particular, how residual SARS-CoV-2 RNA affects signalling pathways in the epipharynx were investigated via spatial gene expression analysis (Visium HD). Moreover, we hypothesize that epipharyngeal abrasive therapy (EAT) targeting the epipharynx could improve long COVID symptoms by modulating local inflammation and gene expression. We conducted a comparative analysis of the gene expression profiles of three patients with long COVID and two control individuals without COVID-19. Residual SARS-CoV-2 RNA was detected in the epipharynx of patients with long COVID, along with the activation of signalling pathways in epithelial and immune cells. After EAT, the viral RNA was either completely cleared or significantly reduced. T-cell receptor signalling pathways were suppressed; the levels of proinflammatory cytokines, such as interleukin-6 and tumour necrosis factor-α, were reduced; and excessive antibody production was mitigated. Histology showed that EAT effectively eliminated the inflamed, dysfunctional ciliated epithelium. This study clarifies that SARS-CoV-2 has long-term effects on the immune response in the epipharynx, emphasizing the need to focus on chronic epipharyngitis as a potential cause of long COVID. Furthermore, EAT may offer a promising approach to alleviating persistent long COVID symptoms.

## Introduction

The epipharynx, located at the posterior part of the nasal cavity (Supplementary Fig. S1), is lined by ciliated epithelium, similar to the nasal cavity, and serves as a crucial target for general upper respiratory tract viral infections. The epipharynx plays a pivotal role in COVID-19 due to the high expression of viral entry factors—angiotensin-converting enzyme 2 (ACE2) and transmembrane protease serine 2 (TMPRSS2)—in the epipharyngeal epithelium^1,2^. The role and susceptibility of this region were underscored during the COVID-19 pandemic. Chronic epipharyngitis, which progresses from acute epipharyngitis caused by viral infection, has been recognized by Japanese otorhinolaryngologists since the 1960s and is characterized by local symptoms such as chronic cough, sore throat, postnasal drip, and pharyngeal discomfort^3^. Chronic epipharyngitis was recently considered to lead to systemic symptoms such as chronic fatigue syndrome due to autonomic nerve regulation disorders and even contribute to immunoglobulin A nephropathy, an autoimmune disease^4–6^. Epipharyngeal abrasive therapy (EAT), which was developed in Japan, is an outpatient treatment method for chronic epipharyngitis. In EAT, the inflamed epipharynx is abraded with a cotton swab soaked in a 1% zinc chloride (ZnCl2) solution, effectively reducing inflammation. EAT has been demonstrated to alleviate local abnormalities in the epipharynx, improve symptoms, and downregulate the mRNA expression of inflammatory cytokines, including interleukin-6 (IL-6) and tumour necrosis factor-alpha (TNFα), in cases of chronic epipharyngitis unrelated to SARS-CoV-2 infection, suggesting a reduction in cytokine secretion^3,7,8^.

There has recently been an increasing focus on persistent inflammation and the residual presence of the virus in the epipharynx as potential factors contributing to the symptoms of long COVID. Long COVID-19 refers to health complications that persist following initial recovery from COVID-19^2^. Such health complications may arise or continue even 1 month after the initial COVID-19 episode, regardless of the severity of the initial episode (asymptomatic, mild, or severe)^9,10^. Studies suggest that 31–69% of COVID-19 survivors experience long COVID symptoms after initial recovery from SARS-CoV-2 infection^11^. Symptoms of long COVID encompass a range of systemic issues, including fatigue, headache, musculoskeletal symptoms, and loss of concentration. Additionally, coughing and dizziness, which are typically observed by otolaryngologists, are also acknowledged as symptoms of long COVID^12–14^. The persistence of viral antigens serves as a foundation for long COVID^15^. Although various treatments have been suggested, most drug therapies only transiently address symptoms, and no medications that target the root cause of the condition are yet available^16^.

One hypothesis is that chronic epipharyngitis plays a role in long COVID, and EAT might alleviate primary symptoms such as fatigue, headache, decreased concentration, and coughing in affected patients^8,17–19^. Our previous findings indicate that although SARS-CoV-2 persists in the epipharyngeal mucosa of patients with long COVID, EAT can effectively remove these antigens, reducing inflammation^8^. It is also hypothesized that chronic tissue inflammation attributed to these residual viral antigens might be part of the aetiology of long COVID^15^. There is further speculation that localized cytokine release eventually induces brain inflammation, leading to long COVID^20^. However, no significant advances have yet been made in local genetic analyses centred on the impacts of these persistent viral antigens on the epipharyngeal immune response.

In the present study, we explored the pathogenesis of long COVID, focusing on the signalling pathways activated by the lingering viral antigens in the epipharynx through spatial gene expression analysis (Visium HD).

## Results

### Assessment of EAT efficacy in patients with long COVID

**Fig. 1:**
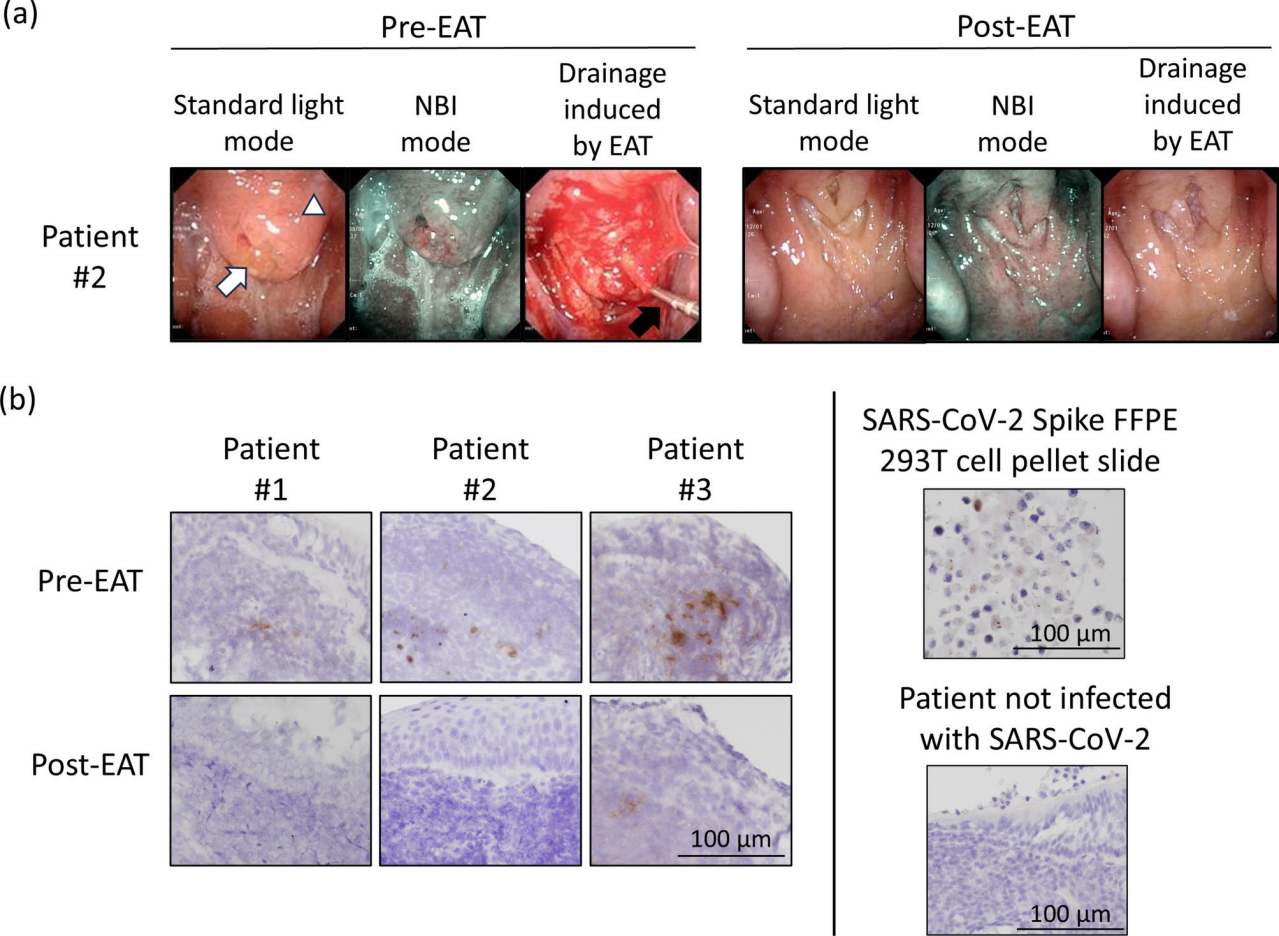
Assessment of Epipharyngeal Abrasive Therapy (EAT) on Macroscopic Epipharyngeal Inflammation and Viral mRNA Expression in Long COVID Patients. (**a**) EAT alleviated the macroscopic epipharyngeal inflammation in long COVID patients. These panels show the endoscopic characteristics in both standard light and narrow-band imaging (NBI) modes and drainage induced by EAT. The left panels show the endoscopic characteristics at the first visit. The right panels show the endoscopic characteristics 3 months after EAT. The white arrowhead indicates swelling of the epipharyngeal mucosa, the white arrow indicates mucus and crust adhesion, and the black arrow indicates a sterile nasal cotton swab containing zinc chloride. (**b**) Expression patterns of SARS-CoV-2 mRNA in the epipharynx in patients with long COVID before and after epipharyngeal abrasive therapy (EAT). The left panels show the staining results of the epipharyngeal tissues before and after EAT in Patients 1 to 3. The right panels show the staining results of the SARS-CoV-2 Spike FFPE 293 T cell pellet slide (GTX435744) as the positive control, and the staining results of epipharyngeal tissue from a patient with chronic epipharyngitis prior to the COVID-19 outbreak as the negative control. Brown dots represent SARS-CoV-2 mRNA. Scale bar, 100 μm.

All patients underwent EAT once a week over a 3-month period. We utilized two main parameters to assess treatment effectiveness: the inflammation score of chronic epipharyngitis and the VAS score for the primary complaint. The endoscopic findings of the epipharynx at the initial visit (pre-EAT) and 3 months after the commencement of EAT (post-EAT) are shown in Fig. 1a, which represents the results for Patient 2. Supplementary Video 1 shows the endoscopic findings of the epipharynx at the initial visit (pre-EAT) during EAT, and Supplementary Video 2 shows the findings 3 months after the commencement of EAT (post-EAT) for Patient 2.

The findings for Patients 1 and 3 are presented in Supplementary Fig. S2. The details of these scores, both pre- and post-EAT, are presented in Table 1. Following EAT, all patients showed significant improvement in their symptoms. The inflammation scores for these patients considerably decreased, and their VAS scores indicated that their primary complaints were alleviated to a point where they no longer faced hindrances in their daily lives.

**Table 1:** Changes in the inflammation scores of chronic epipharyngitis and VAS scores by EAT in patients with long COVID.

Patient number	Inflammation score of chronic epipharyngitis										VAS score	
	Pre-EAT					Post-EAT					First visit	Following 3 months
	Redness	Swelling	Mucus/crust	Bleeding during abrasion	Total	Redness	Swelling	Mucus/crust	Bleeding during abrasion	Total		
1	1	1	1	2	5	0	0	0	1	1	7.5	0
2	2	2	2	2	8	0	0	0	0	0	7	0.2
3	2	1	1	2	6	0	0	0	0	0	8.5	2.9

VAS, visual analogue scale; EAT, epipharyngeal abrasive treatment.

### EAT reduced the levels of residual SARS-CoV-2 RNA in the epipharynx

At the initial consultation, the presence of residual SARS-CoV-2 spike RNA in the epipharynx was confirmed in all three patients, each with varying infection timelines, via in situ hybridization (Fig. 1b). After the administration of EAT once a week for 3 months, the expression of viral RNA disappeared in patients 1 and 2. Patient 3 presented a substantial reduction in viral RNA expression; although complete clearance was not achieved, a marked decrease was observed.

### Spatial gene expression analysis of the epipharynx in long COVID

**Fig. 2:**
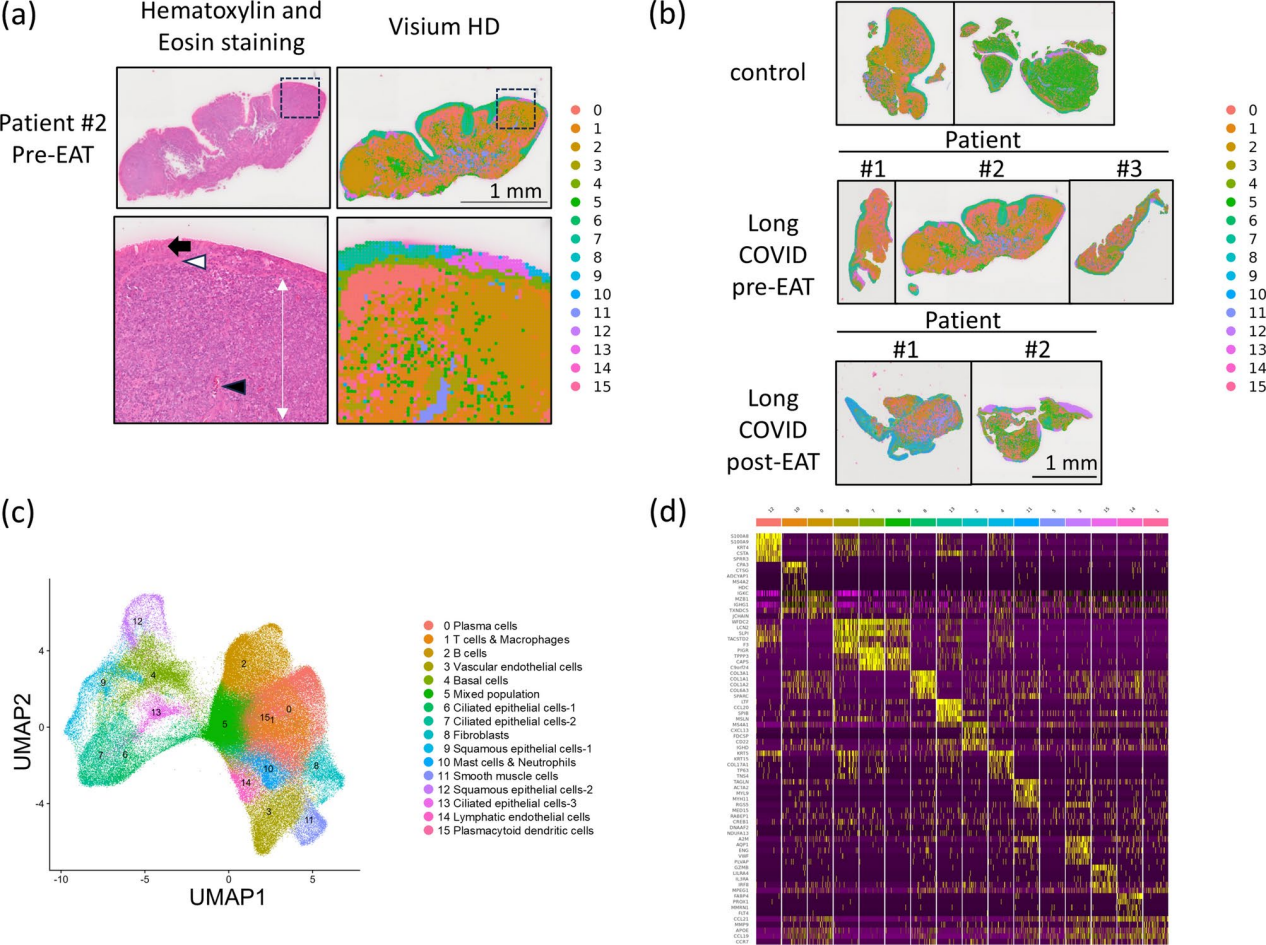
Clustering and gene expression analysis of epipharyngeal cells in control and long COVID samples. (**a**) The left panel shows H&E staining of the epipharyngeal tissue, whereas the right panel displays a clustering map generated via Visium HD on the same region. The upper panels present a low-magnification view, with the dotted box indicating the area that is further magnified in the lower panels. This highlights the high resolution of Visium HD, which enables gene expression analysis at a near single-cell scale. The Visium HD platform achieves a resolution of 8 µm per bin, allowing detailed mapping of gene expression patterns within the complex structure of the epipharyngeal tissue. The black arrow indicates the epithelial region, the white arrowhead indicates the basement membrane, the white double-headed arrow indicates the submucosal region rich in immune cells, and the black arrowhead indicates a blood vessel. This capability facilitates the distinction between epithelial and subepithelial regions and enables comparative analysis of various immune cell populations present in the epipharynx. The cells from the epipharyngeal sample were classified into 16 clusters and mapped onto the tissue. (**b**) Cells from epipharyngeal samples, including control (n = 2), long COVID pre-EAT (n = 3), and long COVID post-EAT (n = 2) samples, were classified into 16 clusters and mapped onto the tissue. (**c**) The 16 clusters are visualized via a UMAP plot with dimension reduction. Each cluster was classified into epithelial clusters and nonepithelial clusters on the basis of their distribution within the tissue. (**d**) Heatmap showing the top 5 DEGs for each of the 16 clusters in the epipharynx.

Spatial transcriptomics analysis was performed using the high-resolution Visium HD Spatial Gene Expression platform (10 × Genomics, Inc., Pleasanton, CA, USA), and the results for Patient 2 before EAT are shown in Fig. 2a. Cluster analysis was conducted to elucidate gene expression patterns in the epipharynx. Using the Seurat package, dimensionality reduction was performed with UMAP for cluster visualization. The analysis identified 16 distinct clusters derived from seven samples: two samples from noninfected individuals, three samples from long COVID patients, and two samples from three long COVID patients post-EAT (Fig. 2b and c). Each cluster was categorized on the basis of gene expression profiles, reflecting different biological characteristics of the epipharynx. The top 5 DEGs for each cluster are presented in the form of a heatmap (Fig. 2d). DEGs other than the top 5 for each cluster are provided in Supplementary File 1. Spatial gene expression analysis was employed to highlight the distribution of cell clusters within the tissue, revealing two major groups of cells on the basis of their spatial distribution: epithelial cells and nonepithelial cells. The epithelial clusters included Clusters 6, 7, 9, 4, 12, and 13. These cells are predominantly composed of nonhaematopoietic cell populations, such as epithelial cells, ciliated cells, and squamous epithelial cells, and are distributed mainly in the epithelial layer. Clusters 6 and 7 presented high expression of genes such as TPPP3, RSPH1, and FOXJ1, indicating a population of ciliated epithelial cells. Clusters 12 and 9 were observed specifically in post-EAT samples, suggesting their involvement in epithelial remodelling and repair processes following therapy. Cluster 4 displayed high expression of basal cell markers such as KRT5, KRT14, and TP63, as well as genes such as COL17A1, LAMA3, and EGFR, indicating that basal cells are involved in regeneration and differentiation. Cluster 13 was characterized by high expression of inflammation-related genes such as LTF and CCL20, suggesting that a cell population is involved in the immune response. The nonepithelial clusters included Clusters 0, 1, 2, 3, 5, 8, 10, 11, 14, and 15 and were primarily composed of immune cells, endothelial cells, dendritic cells, and stromal cells. Cluster 0 presented high expression of genes related to plasma cells, indicating a population of antibody-secreting cells. Cluster 2 was characterized by high expression of B-cell-specific markers, suggesting a population of B cells. Cluster 1 displayed high expression of T-cell-related genes, as well as macrophage-related genes, indicating a mixed population of T cells and macrophages. Cluster 3 exhibited high expression of VWF and other endothelial cell-specific genes, indicating vascular endothelial cells. Cluster 5, which lacked distinctive gene expression patterns, suggested a mixed population of various immune cells and supporting cells. Cluster 8 exhibited high expression of collagen-related genes, indicating fibroblasts. Cluster 10 demonstrated high expression of CPA3 (a mast cell marker) and CTSG (a neutrophil marker), suggesting the presence of mast cells and neutrophils. Cluster 11 displayed high expression of ACTA2 and other smooth muscle-specific genes, suggesting smooth muscle cells. Cluster 14 was characterized by high expression of PROX1, a marker of lymphatic endothelial cells, whereas Cluster 15 was characterized by high expression of genes characteristic of plasmacytoid dendritic cells (pDCs).

### Activation of the SARS-CoV-2 signalling pathway and impairment of ciliary function in the epipharyngeal epithelium in patients with long COVID

**Fig. 3:**
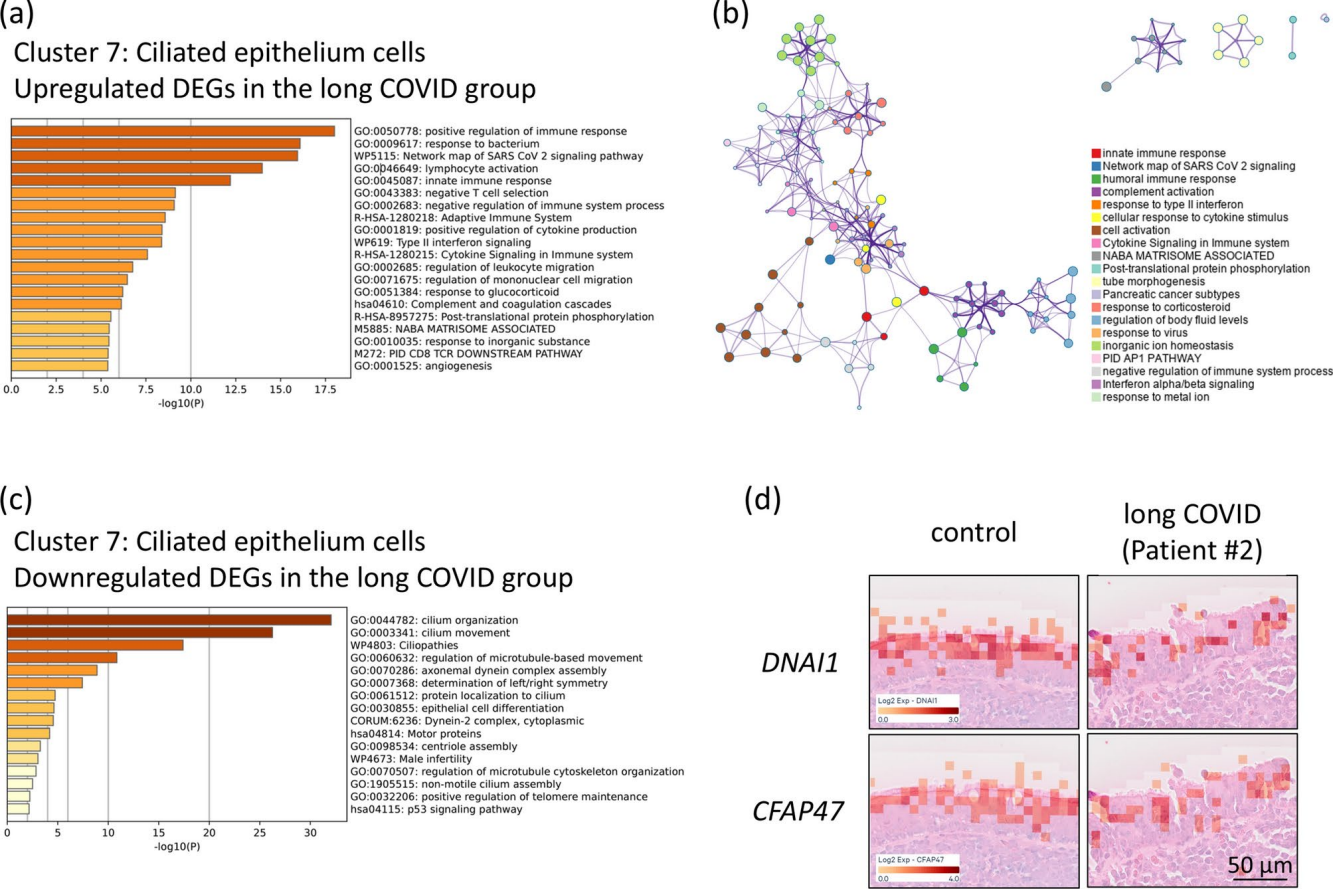
Differential Gene Expression of Epithelial Cell Clusters in the Epipharynx between the Control and Long COVID Groups. (**a**) Bar graph of the enriched GO terms in the upregulated DEGs in epipharyngeal epithelial cells (Cluster 7) of the long COVID group compared with the control group. (**b**) Network of the enriched terms in the upregulated DEGs in the epipharyngeal epithelial cells (Cluster 7) of the long COVID group compared with the control group. The top 20 clusters were selected and rendered as a network, in which terms with a similarity score > 0.3 are connected by an edge. The thickness of the edge represents the similarity score. (**c**) Bar graph of the enriched terms in the downregulated DEGs in the epipharyngeal epithelium cells (Cluster 7) of the long COVID group compared with the control group. (**d**) Spatial gene expression analysis was used to map the expression levels of the Dynein Axonemal Intermediate Chain 1 (DNAI1) and Cilia and Flagella-Associated Protein 47 (CFAP47) genes onto the epipharyngeal tissue in both the control group and the long COVID group. Scale bar, 50 µm.

A comparative gene expression analysis was conducted on the epipharyngeal epithelium (Cluster 7) of three patients with long COVID and two control individuals without COVID-19 to identify DEGs. Gene ontology and pathway enrichment analysis via Metascape revealed that the network map of SARS-CoV-2 signalling (WP5115) was activated in the epipharyngeal epithelia of patients with long COVID (Fig. 3a). This finding suggests the possibility of persistent viral activity in the epipharyngeal tissue, potentially contributing to prolonged symptoms and inflammatory responses associated with long COVID. Furthermore, the network map of SARS-CoV-2 signalling was interconnected with other key immune pathways, including cytokine signalling in the immune system (R-HSA-1280215) and response to virus (GO:0,009,615) (Fig. 3b). These interconnected networks suggest that the persistent immune activation observed in the epipharynxes of patients with long COVID may be initiated by SARS-CoV-2 as the trigger of broader immune responses, contributing to chronic inflammation and prolonged symptoms. SARS-CoV-2 infection induces inflammation and leads to ciliary dysfunction and abnormal ciliary activity^21^. Consistently, there was a decline in pathways related to cilium organization (GO:0,044,782) and cilium movement (GO:0,003,341) in the epipharyngeal epithelium (Fig. 3c). Histological analysis via H&E staining also confirmed ciliary structural damage in the epipharynx in the long COVID group, with significantly lower expression of Dynein Axonemal Intermediate Chain 1 (DNAI1) (adjusted *p* = 9.73E-61) and Cilia and Flagella Associated Protein 47 (CFAP47) (adjusted *p* = 7.23E-49) than in the control group, both of which are involved in ciliary function (Fig. 3d). These results suggest that long-term functional impairment of the epipharyngeal epithelium persists in patients with long COVID. The observed dysregulation of immune and ciliary pathways highlights the complex pathophysiology of long-term COVID, potentially contributing to the persistence of symptoms in affected individuals.

### Increased activation of SARS-CoV-2-related pathways in immune cells in the epipharynx in patients with long COVID

**Fig. 4:**
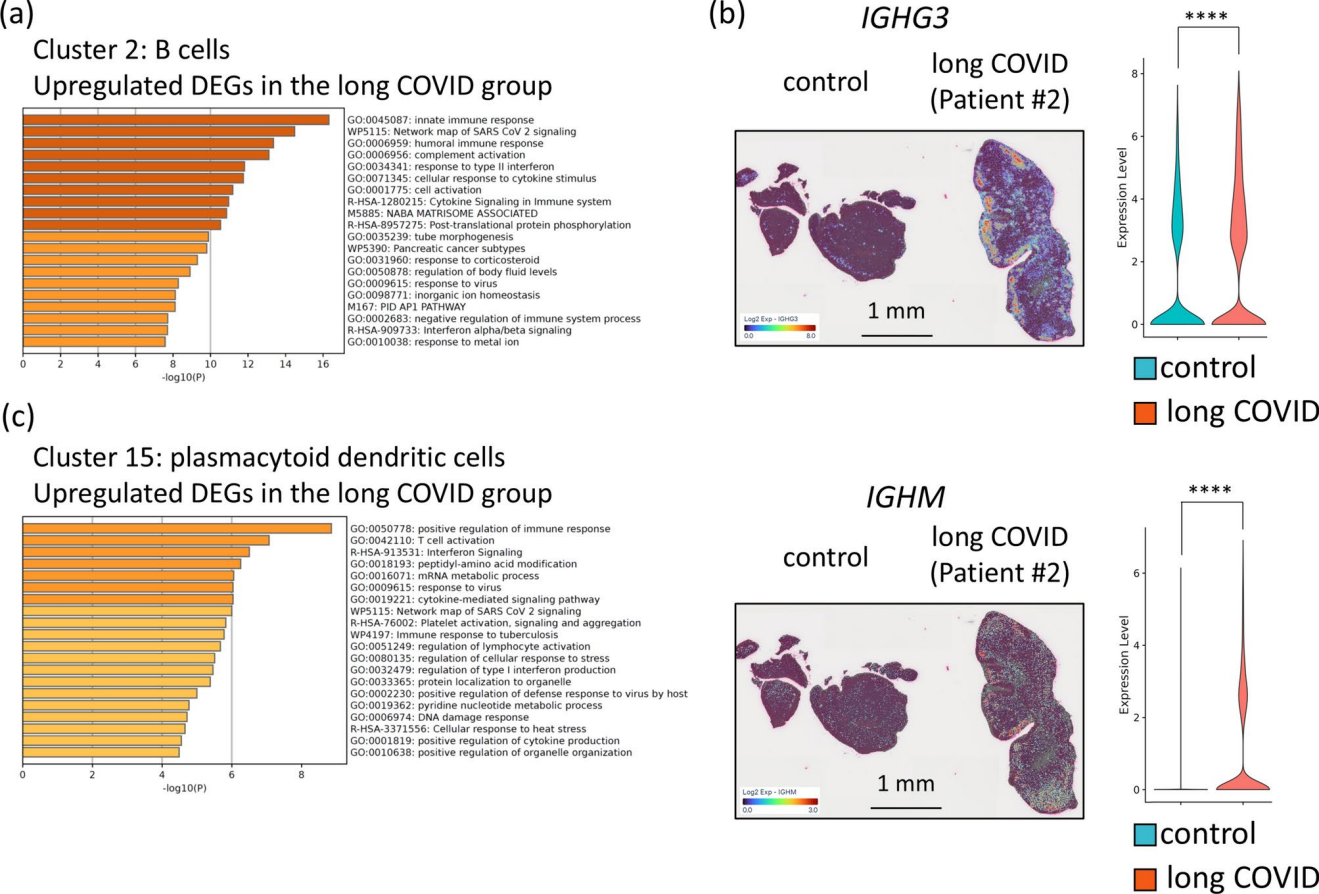
Differential gene expression in the immune cell clusters in the epipharynx between the control and long COVID groups. (**a**) Bar graph of the enriched terms in the upregulated DEGs in the B cells (Cluster 2) of the long COVID group compared with the control group. (**b**) Changes in IGHG3 and IGHM gene expression in the epipharynx between the control and long COVID groups. The left panels show spatial gene expression analysis mapping the expression levels of the IGHG3 and IGHM genes onto the epipharyngeal tissue. The right panels show violin plots depicting the expression levels of these two genes. Blue represents data from the control group, whereas red indicates data from the long COVID group. ***p < 0.0001. (**c**) Bar graph of the enriched terms in the upregulated DEGs in the plasmacytoid dendritic cells (cluster 15) of the long COVID group compared with the control group.

Gene expression analysis of B cells (Cluster 2) from patients with long COVID revealed activation of the network map of SARS-CoV-2 signalling (WP5115). This activation suggests a sustained immune response against residual viral antigens in patients with long COVID. In addition, pathways related to cell activation (GO:0,001,775) and positive regulation of the immune response (GO:0,050,778) were also enriched in the upregulated DEGs in B cells, indicating an enhanced immune response (Fig. 4a). This heightened activity in B cells may lead to the activation and differentiation of plasma cells. Indeed, gene expression analysis of the plasma cell cluster (Cluster 0) revealed significantly higher expression levels of IGHG3 and IGHM in patients with long COVID than in the control group (Fig. 4b). These findings imply persistent immune activation and chronic inflammatory responses in patients with long COVID. Moreover, in the plasmacytoid dendritic cells (pDCs) (Cluster 15) of patients with long COVID, several pathways, including positive regulation of the immune response (GO:0,050,778) and interferon signalling (R-HSA-913531), were significantly enriched. Pathways related to the response to viruses (GO:0,009,615) and the network map of SARS-CoV-2 signalling (WP5115) were also enriched (Fig. 4c). These findings suggest that pDCs continue to recognize and respond to viral components, triggering excessive immune responses and maintaining a chronic inflammatory state in the upper pharynxes of patients with long COVID. The activation of these pathways in pDCs, together with the heightened responses of B cells and plasma cells, indicates that a complex network of interactions contributes to prolonged inflammation and immune dysregulation in patients with long COVID. Although no significant pathways (q < 0.01) were detected in T cells (Cluster 1), several immune-related pathways were enriched, including the regulation of the MAPK cascade (GO:0,043,408) and the binding of TNFs to their physiological receptors (R-HSA-5669034) (Supplementary Fig. S3). These findings suggest that T cells still play a subtle role in the immune dysregulation observed in patients with long COVID. Collectively, these findings indicate that interactions among pDCs, B cells, plasma cells, and T cells contribute to the chronic inflammation and immune dysregulation characteristic of patients with long COVID, highlighting the complex and multifaceted nature of the immune response in this condition.

### Epipharyngeal abrasive therapy (EAT) eliminates infection-induced dysfunctional ciliated epithelium in long COVID patients

**Fig. 5:**
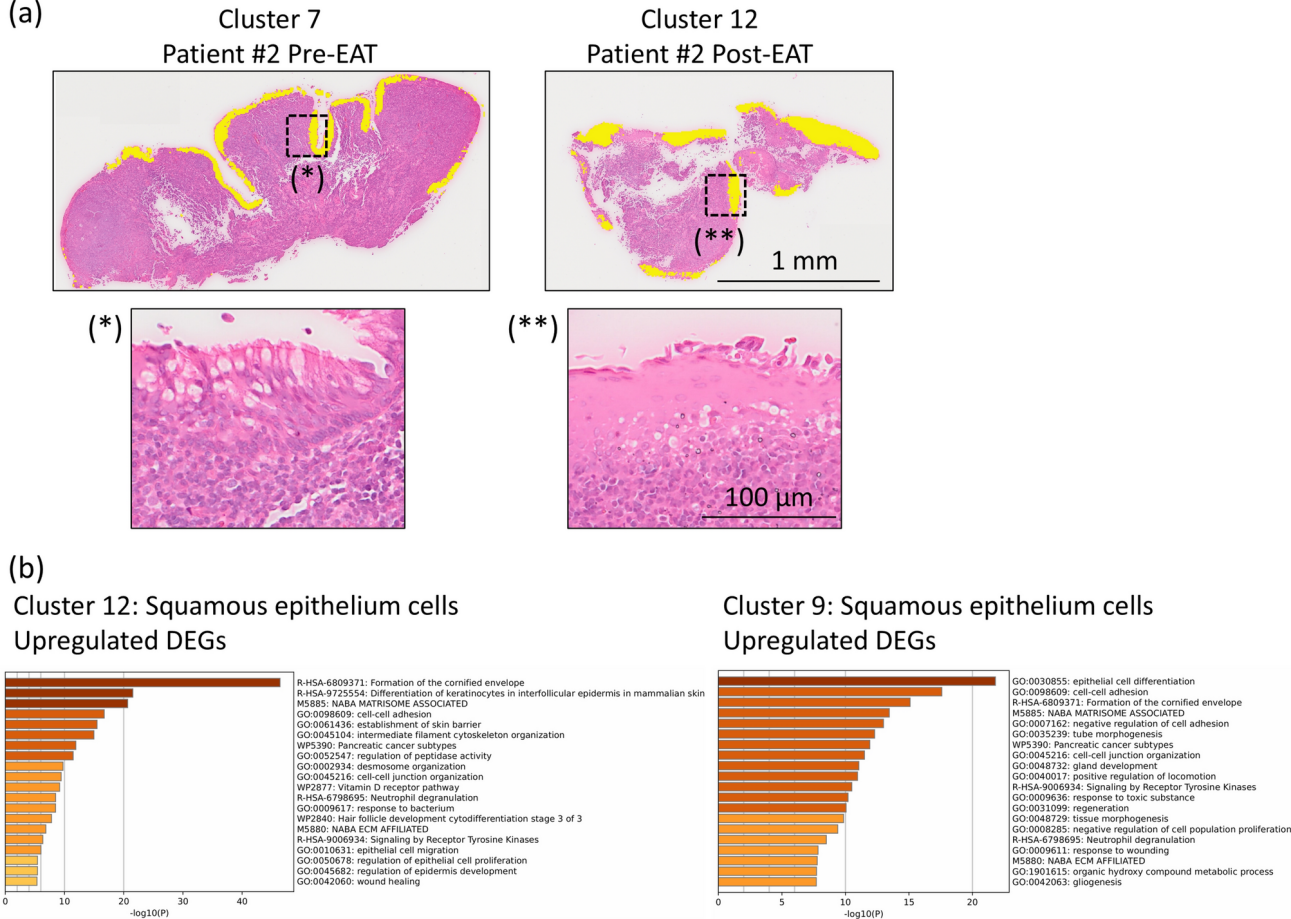
Spatial gene expression and histological analysis of the epipharyngeal epithelium before and after treatment in patients with long COVID. (**a**) The upper panels show the spatial gene expression analysis of the epipharyngeal tissue in long COVID patients pre-EAT and post-EAT. The epithelium pre-EAT showed a higher proportion of Cluster 7, whereas the epithelium post-EAT showed higher proportions of Clusters 12 and 9, each of which is highlighted in yellow in the tissue. The lower panels display H&E staining results with magnified views of the epithelium, highlighting structural changes before and after treatment. (**b**) Bar graph of the enriched terms in the upregulated DEGs in Clusters 12 and 9, representing the post-EAT epithelium.

In the pre-EAT state of long COVID Patient 2, the tissue surface was predominantly covered by ciliated epithelium (Cluster 7). In contrast, following treatment, the ciliated epithelium was no longer observed, and a new cluster of squamous epithelial cells (Cluster 12) emerged, which was confirmed by histological analysis (Fig. 5a). Enrichment analysis of the newly emerged cluster (Cluster 12 in Patients 2 and 9 in Patient 1), which appeared on the apical surface, was performed using the top 300 DEGs and revealed the activation of pathways associated with the squamous epithelium, including epidermis development (GO:0,008,544), differentiation of keratinocytes in the interfollicular epidermis in the establishment of the skin barrier (GO:0,061,436) and formation of the cornified envelope (R-HSA-6809371) (Fig. 5b). These findings suggest that squamous metaplasia occurred at the genetic level. Collectively, these results demonstrate that EAT effectively eliminates the inflamed ciliated epithelium in patients with long COVID and induces the formation of squamous cells with high barrier function.

### Modulation of T-cell receptor pathways, inflammatory cytokines, and IGHM expression in the epipharynxes of patients with long COVID-via EAT

**Fig. 6:**
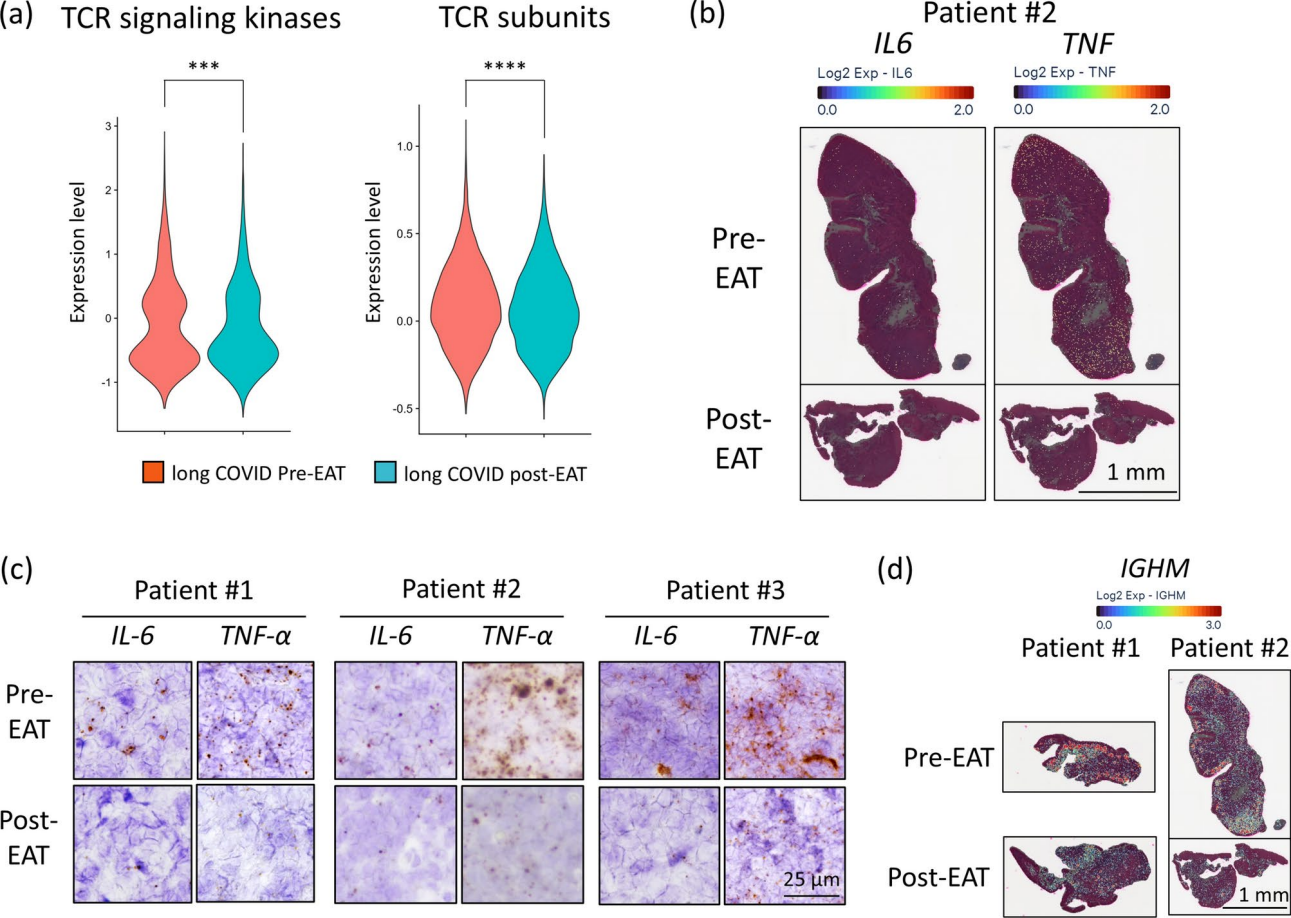
Changes in TCR Signalling, Inflammatory Cytokine Expression, and IGHM Gene Expression in Long COVID Patients Pre- and Post-EAT. (**a**) Comparison of T-cell receptor (TCR) signalling kinase and TCR subunit expression levels in cluster 1 between the long COVID pre-EAT group and the post-EAT group. The panels show violin plots depicting the scores calculated on the basis of the expression levels of TCR signalling kinases and TCR subunits from the network map of SARS-CoV-2. Red represents long COVID pre-EAT and blue represents long COVID post-EAT. ***p < 0.001. ****p < 0.0001. (**b**) Spatial gene expression analysis was used to map the expression levels of the IL-6 and TNF genes onto the epipharyngeal tissue in both pre-EAT and post-EAT samples, highlighting the spatial distribution of these inflammatory markers. Scale bar, 1 mm. (**c**) These panels depict the mRNA expression patterns of IL-6 and TNF-α in the subepithelial region of the epipharyngeal tissues from patients with long COVID as determined by in situ hybridization. Each row represents a different patient (Patients 1 to 3), with the left and right panels showing IL-6 and TNF-α expression, respectively. The top row of images shows the expression pre-EAT, whereas the bottom row shows the expression post-EAT. The brown dots represent IL-6 and TNF-α mRNA. Scale bar, 25 μm. (**d**) Spatial gene expression analysis maps the expression levels of IGHM genes onto the epipharyngeal tissue in both pre-EAT and post-EAT samples, highlighting the spatial distribution of *IGHM*. Scale bar, 1 mm.

EAT was found to suppress both T-cell receptor (TCR)-related pathways and downregulate inflammatory cytokine expression in the epipharynxes of long COVID patients. Analysis of the “Network Map of SARS-CoV-2 (WP5115)” revealed that the activation of TCR signalling cascades (TCR signalling kinases and TCR subunits) in the T-cell population of Cluster 1 was inhibited following EAT (Fig. 6a). IL-6, which is known to promote the proliferation of T cells, and TNF-α, which is released from T cells to exert antiviral effects, were both downregulated after EAT. Spatial gene expression analysis revealed a decrease in the expression of inflammation-related cytokines in both Patient 1 and Patient 2 after treatment. Patient 2’s results are shown as a representative in Fig. 6b, whereas the results for Patient 1 are presented in Supplementary Fig. S4. Furthermore, all patients showed a reduction in the expression of IL-6 and TNFα in situ after EAT (Fig. 6c). Spatial gene expression analysis revealed a decrease in the expression of IGHM after treatment in Patients 1 and 2 (Fig. 6d). Indeed, gene expression analysis of the plasma cell cluster (Cluster 0) revealed significantly lower expression levels of IGHM in the long COVID post-EAT group than in the long COVID pre-EAT group (*p* < 0.001). These findings suggest that EAT may effectively reduce excessive activation of TCR-related pathways, persistent inflammation, and overactivation of humoral immune responses in the epipharynx, potentially contributing to the clinical improvements observed in patients with long COVID.

## Discussion

In this study, we evaluated the relationship between long COVID and chronic epipharyngitis, as well as the effectiveness of epipharyngeal abrasive therapy (EAT). Although previous reports have demonstrated the clinical association between long COVID and chronic epipharyngitis, as well as the effectiveness of EAT in alleviating symptoms^8,17,18^, there is a lack of fundamental research-based evidence to support these findings. To address this gap, we conducted spatial gene expression analysis using Visium HD technology to elucidate the molecular mechanisms by which EAT contributes to symptom relief. This innovative approach allowed us to precisely map gene expression changes in the epipharynx, offering a comprehensive view of how SARS-CoV-2 affects the tissue microenvironment at the cellular level.

Insights into the immunobiology of long COVID underscore the potential role of residual viral particles, postviral autoimmune disorders, and unresolved tissue damage as a result of chronic inflammation^22,23^. Our study provides important evidence supporting these hypotheses by showing that SARS-CoV-2 spike RNA- and SARS-CoV-2-related inflammation persist in the epipharynx for more than six months post infection. Although no expression of the SARS-CoV-2 spike protein in the epipharynx was evident in these patients with long COVID (data not shown), genetic analysis revealed activation of WP5115, the network map of the SARS-CoV-2 signalling pathway (https://www.wikipathways.org/instance/WP5115) in epithelial cells, B cells, and pDCs. This aligns with theories that even nonactive viral fragments may possess biological significance^24^. This persistence of inflammation, identified for the first time at the genetic level, highlights the epipharynx as a critical site for continual immune responses in long COVID patients, suggesting that residual viral antigens may contribute to the chronic symptoms experienced by these patients. Furthermore, our study revealed that IGHG3 and IGHM levels are significantly elevated in the plasma cells of patients with long COVID, even beyond the initial infection phase^25^. This persistent immune activation and antibody production suggest a role in the ongoing symptoms and multiorgan involvement observed in long COVID patients. Moreover, the continued presence of active plasma cells also demonstrates that the epipharynx is a crucial site for sustained immune responses, providing new insights into the prolonged symptoms experienced by these patients.

SARS-CoV-2 infection commonly starts in the upper respiratory tract, frequently resulting in localized symptoms such as cough, nasal obstruction, and sore throat, particularly in mild to moderate cases^26,27^. The substantial involvement of the upper respiratory tract, especially the epipharyngeal region (a preferred site for SARS-CoV-2 diagnostic testing^28^), indicates its pivotal role during acute infection. It is therefore plausible that the residual effects in this region contribute to persistent long COVID symptoms. Notably, chronic epipharyngitis is found in 90% of patients with long COVID^18,29,30^. The fact that most of these patients initially present with mild acute-phase symptoms ^23^ underscores a crucial connection between initial COVID-19 symptoms and the development of chronic epipharyngitis within the context of long COVID. This association suggests a pathophysiology wherein even mild initial infections may have substantial and persistent implications.

In patients responsive to EAT, we observed reduced epipharyngeal inflammation, which is consistent with previous studies on the benefits of EAT in patients with long COVID^17,18,29^. Elevated secretion of the proinflammatory cytokines IL-6 and TNF-α has been documented in patients with long COVID^31,32^. EAT may improve symptoms of an infection that is confined to the upper respiratory tract, by reducing cytokine secretions in the epipharynx. Recent studies suggest that the encephalopathy associated with COVID-19 is not a result of direct viral proliferation within the brain but arises from indirect causes^20^. Specifically, inflammatory cytokines produced in the lungs secondary to transfection of the SARS-CoV-2 gene by a virus vector nasally administered to mice induced inflammation in the brain. Furthermore, mice under these conditions presented symptoms of malaise, depression, and brain inflammation. The potential of proinflammatory cytokines to traverse the blood–brain barrier and induce brain damage adds another layer to understanding the pathology and neurological manifestations of long COVID^33^. Additionally, the elevated proinflammatory cytokine levels in the epipharynx and the potential systemic effects add another dimension to understanding the diverse symptoms of long COVID.

A notable finding in this study was the modulation of T-cell receptor (TCR) signalling pathways and inflammatory cytokine expression in the epipharynx of patients with long COVID by EAT treatment. Long COVID-19 is associated with sustained activation of SARS-CoV-2-specific T cells, characterized by a stable effector phenotype and key TCR signatures postinfection, indicating persistent TCR signalling and robust T-cell responses^34^. Persistent activation of T cells in long COVID may contribute to chronic immune activation^35^. Specifically, TCR signalling pathways involving kinases and TCR subunits was activated in pre-EAT samples and suppressed following EAT. The observed suppression of these pathways after EAT suggests that EAT may help restore immune balance in the epipharynx by modulating T-cell activity, thereby reducing the chronic inflammatory state observed in patients with long COVID.

In addition to its effect on TCR signalling, EAT significantly reduces the expression of inflammatory cytokines, such as interleukin-6 (IL-6) and tumour necrosis factor-α (TNF-α), which are closely linked to the pathophysiology of long COVID^36^. Elevated levels of these cytokines have been associated with persistent inflammation, systemic symptoms, and immune dysregulation in patients with long COVID^31,37^. Interestingly, the activation of TCR signalling is known to promote the release of proinflammatory cytokines such as IL-6 and TNF-α, further perpetuating the inflammatory response and contributing to the persistence of symptoms^38^. The reduction in IL-6 and TNF-α expression observed in this study indicates that EAT may attenuate the inflammatory response not only through direct modulation of T-cell activity but also by inhibiting the cytokine-mediated feedback loop that sustains chronic inflammation. Collectively, these findings suggest that EAT has a comprehensive therapeutic effect on long COVID by simultaneously modulating TCR signalling pathways and reducing the expression of key inflammatory cytokines. This dual action may contribute to alleviating both local and systemic symptoms in patients with long COVID, highlighting the potential of EAT as a therapeutic intervention that addresses the underlying immune dysregulation and inflammation in this condition. Moreover, our study revealed that EAT not only modulates T-cell activity but also suppresses the humoral immune response by downregulating IGHM expression in plasma cells. These findings suggest that EAT may inhibit excessive antibody production, which is another critical factor in the persistence of long COVID symptoms. This dual effect on both cellular and humoral immunity highlights the comprehensive role of EAT in alleviating chronic inflammation in long COVID.

EAT demonstrated great therapeutic potential by clearing residual viral RNA and reducing the expression of inflammatory cytokines in the epipharynx. This reduction in inflammation was accompanied by the elimination of inflamed, dysfunctional ciliated epithelial cells and the induction of squamous metaplasia, which may contribute to restoring the barrier function of the epipharyngeal mucosa. The histological changes induced by EAT have been demonstrated in previous studies^1^. Similarly, in laser treatment for nasal allergies, fibrotic tissue with reduced inflammatory cell infiltration forms in the soft tissue of the inferior turbinate, contributing to reduced inflammation in ciliated epithelium^39^. This histological alteration temporarily prolongs mucociliary transport, with a return to near preoperative levels observed by the 3-month follow-up^40^. A similar recovery process is anticipated with EAT. Additionally, adenoidectomy, a surgical procedure that physically removes the pharyngeal tonsils in the epipharynx, does not negatively impact cellular or humoral immunity^41^. Given that EAT also targets the same region, it is expected that EAT similarly does not adversely affect immune function. The efficacy of EAT could be attributed to multiple factors, including the mechanical removal of inflamed tissue and the anti-inflammatory effects of zinc chloride (ZnCl2). ZnCl2 is known to inhibit the replication of SARS-CoV-2 by targeting its main protease and limiting viral activity in vitro^42^. Additionally, ZnCl2 has been reported to induce protein denaturation and reduce exudate from treated areas, contributing to its anti-inflammatory properties^43,44^. We consider that while ZnCl₂ contributes to the anti-inflammatory effects in chronic epipharyngitis, and the removal of residual viral RNA is primarily influenced by the mechanical abrasion during EAT. However, to further clarify the specific role of ZnCl₂, we are planning a multi-institutional study comparing the efficacy of EAT with or without the use of ZnCl₂. This study will allow for a more precise evaluation of the contribution of ZnCl₂ to the therapeutic effects of EAT. Importantly, EAT has been shown to reduce the expression of IL-6 and TNF-α in the epipharynx not only in patients with long COVID but also in those with chronic epipharyngitis unrelated to SARS-CoV-2 infection^7^. These findings suggest that the therapeutic benefits of EAT are dependent not only on the removal of viral antigens but also on the broad anti-inflammatory effects of ZnCl2 on the epipharyngeal mucosa. These findings highlight the potential of EAT as a versatile therapeutic approach for various inflammatory conditions of the epipharynx. A recent report indicated that ZnCl2 used in EAT lowers the exhaled nitric oxide level, which is correlated with the severity of epipharyngitis; this finding supports the anti-inflammatory effects of ZnCl2 on the epipharyngeal mucosa^45^. A severe immune response may underpin the autonomic dysfunction observed in patients with long COVID and may be linked to their myriad symptoms^46^, including dizziness, tachycardia, fluctuating blood pressure, headaches, chronic cough, “brain fog,” and reduced exercise tolerance, which are indicative of an imbalanced autonomic system^47,48^. EAT may also influence the autonomic nervous system, as some reports suggest its potential to modulate autonomic function^5^, thereby reducing symptoms such as dizziness and chronic fatigue. By combining these properties, EAT may effectively reduce inflammation, alleviate symptoms, and improve overall quality of life in patients with long COVID.

Our study has three key features. First, state-of-the-art Visium HD spatial gene expression analysis was utilized to address the global issue of long COVID by providing a detailed understanding of its pathophysiology at the cellular level. Second, we are the first to demonstrate that SARS-CoV-2-related inflammation persists in the epipharynx of patients with long COVID over an extended period, contributing to the chronic symptoms observed. Third, we have shown that EAT can effectively modulate key signalling pathways and cytokine expression, providing a potential therapeutic approach for managing long COVID symptoms. These findings underscore the need for further research into the specific mechanisms of EAT and its broader applications in long COVID management.

Our study has several limitations. The greatest limitation is the small sample size, which may affect the generalizability of our findings. However, by utilizing state-of-the-art Visium HD spatial gene expression analysis, we were able to obtain highly detailed and comprehensive data from each individual case, providing robust insights into the molecular mechanisms underlying long COVID. This advanced technology allows us to overcome some of the constraints typically associated with small sample sizes by offering a more precise and in-depth analysis of the cellular and molecular changes in the epipharynx.

Another limitation is that long COVID encompasses a wide range of symptoms, including those beyond upper respiratory tract symptoms, and determining which specific symptoms respond to EAT requires large-scale analysis. Currently, the evidence supporting the effectiveness of EAT in long COVID treatment is limited to reports from individual facilities. To address this issue, a multicentre collaborative study is currently being conducted by the Epipharyngeal Abrasive Therapy Review Committee of the Japan Society of Stomato-pharyngology. This study aims to evaluate the efficacy of EAT across multiple facilities, providing valuable data that will help clarify the therapeutic potential of EAT and its application in long COVID management. The results of this research are eagerly awaited to gain a more comprehensive understanding of the effectiveness of EAT and to establish more robust evidence for its use in clinical practice.

Despite these limitations, our study provides important clinical insights, suggesting a potential association between long COVID and chronic epipharyngitis and highlighting the therapeutic potential of EAT in managing long COVID symptoms. Future studies with larger sample sizes and collaborative efforts across multiple institutions will be crucial in confirming these findings and elucidating the underlying mechanisms by which EAT treats long COVID. Such research could provide more definitive evidence regarding the role of the epipharynx in the pathophysiology of long COVID and help establish standardized protocols for the use of EAT in clinical practice.

## Methods

### Patients and tissue samples

Patient data were obtained with permission from the Ethics Committee of Fukuoka Dental College (ID: 552). Tissue samples were obtained via endoscopic epipharyngeal biopsy from February 2021 to February 2023. These samples were obtained using biopsy forceps specifically designed for endoscopy (model VDK-FB-18-105-O-P-A1P; PENTAX Medical, Tokyo, Japan). Samples were collected from three patients who developed chronic epipharyngitis prior to December 2019, when COVID-19 was first reported and from three patients who were confirmed to be infected with COVID-19 during the Omicron-dominant period in Japan and underwent EAT. All participants provided written informed consent. The research was conducted in accordance with the Declaration of Helsinki and Title 45, US Code of Federal Regulations, Part 46, Protection of Human Subjects, effective as of 13 December 2001.

### Patients’ baseline characteristics

We evaluated three patients at our EAT-specialized outpatient unit situated within a university hospital. Each patient had persistent symptoms since their initial COVID-19 diagnosis, prompting them to seek EAT care. The detailed characteristics of each patient are summarized in Table 2. Patient 1, a 45-year-old woman, was diagnosed with a mild presentation of COVID-19 in August 2022. One month after her initial infection, she was hospitalized for chronic dizziness. During her hospitalization, cranial magnetic resonance imaging revealed no intracranial lesions. She then visited our unit 2 months postinfection because of the continued dizziness. Patient 2, a 21-year-old man, consulted with us 4 months after the onset of COVID-19 in April 2022; his chief complaint was persistent cough. (This patient was described in a previous case study^8^). Patient 3, a 24-year-old man, visited 6 months postinfection in May 2022 because of persistent severe fatigue that prevented him from attending college. Among the cohort, only Patients 2 and 3 had received COVID-19 vaccination before their respective infections. All three patients underwent nasal irrigation as self-care for chronic epipharyngitis. All patients contracted COVID-19 during the Omicron-dominant period in Japan^49^.

**Table 2:** Clinical details of patients.

Patient number	Age (y)	Sex	Period from infection to start of EAT (months)	Chief complaint	Date of confirmed COVID-19 positivity	Severity of COVID-19	Medical history	BMI, kg/m^2^	Number of vaccinations before infection	Number of vaccinations after infection	Treatment other than EAT
1	45	F	2	Dizziness	08/2022	Mild	None	18.1	0	0	Nasal irrigation
2	21	M	4	Cough	04/2022	Mild	Chronic sinusitis	20.8	2	0	Nasal irrigation Low-dose macrolides Carbocisteine Mometasone furoate
3	24	M	6	Fatigue	05/2022	Mild	None	24.6	3	0	Nasal irrigation

M, male; F, female; EAT, epipharyngeal abrasive therapy; COVID-19, long-term coronavirus disease 2019; BMI, body mass index.

### EAT

EAT is a treatment method for chronic epipharyngitis that involves the use of a medical swab with an aluminium shaft (AL1503RY-50, Heiwa Medic, Takayama, Gifu, Japan) soaked in 1% ZnCl2 solution, as described previously^2,7^. EAT was performed in the outpatient department once a week by a board-certified otorhinolaryngologist. A 1% ZnCl2 solution was prepared in-house and used with the approval of the Clinical Ethics Committee of Fukuoka Dental College Hospital.

### Inflammation score of chronic epipharyngitis and visual analogue scale (VAS) score

The inflammation score of chronic epipharyngitis was calculated according to the following four assessment criteria established by the Japan Society of Stomato-pharyngology EAT Review Committee: redness of the epipharyngeal mucosa, swelling of the epipharyngeal mucosa, mucus or crust adhesion, and bleeding during abrasion. Each criterion was rated on a 3-point scale (0: none, 1: mild/moderate, or 2: severe). The VAS score for the primary complaint ranged from 0 (absence of symptoms) to 10 (highest severity of symptoms).

### *RNA* in situ *hybridization staining*

Tissues were fixed in 10% neutral buffered formalin and embedded in paraffin, and the resulting tissue blocks were then cut into 4-μm-thick sections for in situ hybridization. To detect interleukin-6 (IL-6), tumour necrosis factor-α (TNF-α), and SARS-CoV-2 spike mRNA in the tissue, an RNA scope (in situ hybridization system; Advanced Cell Diagnostics, Newark, CA, USA; IL-6: No. 310371, TNF-α: No. 310421, and SARS-CoV-2: No. 848561) was used in accordance with the manufacturer’s guidelines. For the positive control of SARS-CoV-2 RNA, we procured the SARS-CoV-2 Spike FFPE 293 T cell pellet slide (GTX435744) from GeneTex, Inc. (Irvine, CA, USA). All images were captured using a microscope (AXIO Vert. A1; Carl Zeiss AG, Oberkochen, Germany).

### Spatial transcriptomics and statistical analyses (Visium HD)

Formalin-fixed paraffin embedded (FFPE) tissues from seven samples were analysed. This included two samples from patients without COVID-19, three samples from patients with long COVID (specifically, Patients 1, 2, and 3 from Table 2), and one sample from two of the patients with long COVID after EAT treatment (specifically, Patients 1 and 2). FFPE tissue sections were cut at a thickness of 5 µm and placed on glass slides. Haematoxylin and eosin (H&E) staining was performed according to the 10 × Genomics protocol. After staining, the slides were imaged at 20 × magnification via a VS200 slide scanner (Evident Corp., Tokyo, Japan). The RNA was transferred to Visium HD slides using the CytAssist. cDNA synthesis and library construction were performed using the Visium HD Library Preparation Kit (10 × Genomics). Briefly, mRNA was captured by barcoded probes, reverse transcribed into cDNA and amplified for sequencing library preparation. Libraries were sequenced on an Illumina NovaSeq X Plus (Illumina, Inc., San Diego, CA, USA) with a 150 × 150 bp paired-end configuration. The sequencing reads were then aligned to the human reference genome (GRCh38) with Space Ranger v3.0 (10 × Genomics, Inc.). The 2 µm bins were merged into 8 µm bins. Then, gene expression matrices were generated. The output count matrices were processed with Seurat (5.1.0) in R^50^. The samples were merged and normalized via log normalization. For dimensionality reduction and unsupervised clustering, the sketch method of Seurat v5 was utilized to improve performance and reduce the calculation cost. First, 50,000 cells were selected on the basis of the leverage score calculated via the ‘SketchData’ function. Principal component analysis and uniform manifold approximation and projection (UMAP) were applied to the subset dataset for visualization. Leiden clustering (resolution = 0.5) was applied to the shared nearest neighbours (dims = 1:50). Dimensionality reduction and clustering results were extended to the full dataset. A heatmap was generated to show the top 5 differentially expressed genes (DEGs) with the lowest adjusted p values for each cluster. DEGs other than the top 5 for each cluster are provided in Supplementary File 1. This file includes comprehensive data on the expression levels and statistical significance of these genes. To identify DEGs between two patients without COVID-19 and three patients with long COVID, the Wilcoxon rank sum test was performed, with an adjusted *p* value of < 0.05. Genes with a fold change (FC) ≥ 1.5 were considered upregulated in the long COVID group, whereas those with a 2/3 > FC were considered downregulated in the long COVID group. The list of DEGs was subsequently input into Metascape for Gene Ontology and pathway enrichment analysis^51^. Spatial gene expression analysis was performed via Loupe Browser software (10 × Genomics, Inc.). Violin plots were created to illustrate the changes in the expression levels of IGHG3 and IGHM between two patients without COVID-19 and three patients with long COVID-19. A detailed list of DEGs between the two patient groups has been compiled and is provided in Supplementary File 2. Additionally, scores that represent specific cascades of the network map of SARS-CoV-2 (WT5115), such as TCR signalling kinases and TCR subunits, were calculated via the ‘AddModuleScore’ function and visualized with violin plots. The genes used for calculating the score were; ‘TCR signaling kinase in PBMC’: *FYN, LCK, and ZAP70*; ‘TCR subunits in PBMC’: *BTN3A1*, *C1QBP*, *CAMK4*, *CARD11*, *CD247*, *CD3E*, *CD3G*, *FYN*, *GP1BA*, *HBB*, *HBD*, *HRG*, *LCK*, *PF4*, *RAC1*, *SERPINE1*, *SKAP1*, *TRAC*, and *TRBC1*. The Wilcoxon rank sum test was used to evaluate the significance of differences between pre- and post-EAT. The p values were adjusted by Bonferroni correction.

## Supplementary Information

Supplementary Information 1.

Supplementary Video 1.

Supplementary Video 2.

Supplementary Information 2.

Supplementary Information 3.

## Data Availability

The spatial transcriptomic data generated in this study are deposited in the EMBL’s ArrayExpress database under the accession number E-MTAB-14669.
