# Supplementary material for: Spatial transcriptomics of the epipharynx in long COVID identifies SARS-CoV-2 signalling pathways and the therapeutic potential of epipharyngeal abrasive therapy
Source: Sci Rep. 2025 Mar 12;15:8618. doi: 10.1038/s41598-025-92908-7 (PMC11903674; doi:10.1038/s41598-025-92908-7)
Supplement: Supplementary file 1 — Supplementary Information 1. [file 41598_2025_92908_MOESM1_ESM.pdf]

Supplementary Information

Spatial Transcriptomics of the Epipharynx in Long COVID Identifies SARS-CoV-2 Signalling Pathways and the Therapeutic Potential of Epipharyngeal Abrasive Therapy

Kensuke Nishi[1,2,3,*,†], Shohei Yoshimoto[2,4,†], Takayuki Tanaka[5], Shoichi Kimura[1,5], Toshiyuki Tsunoda[6], Akira Watanabe[7,*], Kaori Teranaka[7], Yo Oguma[7], Hanako Ogawa[7], Takumi Kumai[8], and Takafumi Yamano[1,2]

[1]Section of Otolaryngology, Department of Medicine, Fukuoka Dental College, Fukuoka, 814-0193, Japan

[2]Oral Medicine Research Center, Fukuoka Dental College, Fukuoka, 814-0193, Japan

[3]Nishi Otolaryngology Clinic, Fukuoka, 814-0031, Japan

[4]Section of Pathology, Department of Morphological Biology, Division of Biomedical Sciences, Fukuoka Dental College, Fukuoka, 814-0193, Japan

[5]Department of Otolaryngology, Faculty of Medicine, Fukuoka University, Fukuoka, 814-0180, Japan

[6]Department of Cell Biology, Faculty of Medicine, Fukuoka University, Fukuoka, 814-0180, Japan

[7]CyberomiX Co., Ltd., Kyoto, 602-8407, Japan

[8]Department of Innovative Head & Neck Cancer Research and Treatment, Asahikawa Medical University, Asahikawa, 078-8510, Japan

Supplementary Figure 1

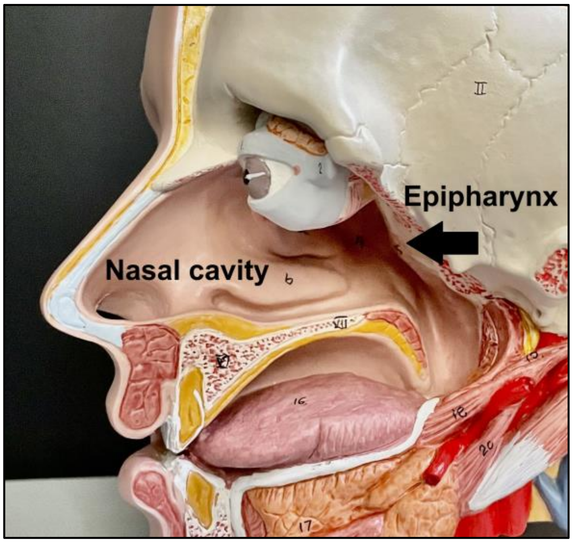

The structure of the epipharynx is illustrated using a 3D model (Nihon 3B Scientific Inc., Niigata, Japan). The black arrow indicates the epipharynx.

Supplementary Figure 2

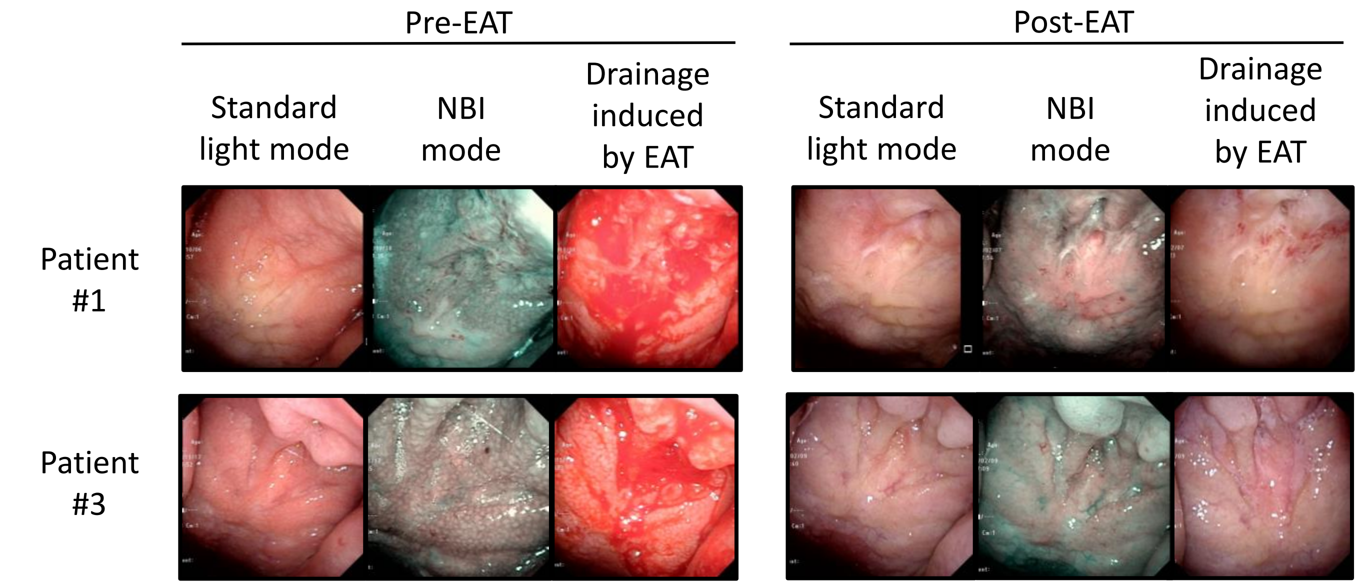

The endoscopic images of the epipharynxes of Patients 1 and 3 before and after 3 months of EAT treatment.

Supplementary Figure 3

Cluster 1: T cells & Macrophages
Upregulated DEGs in the long COVID group

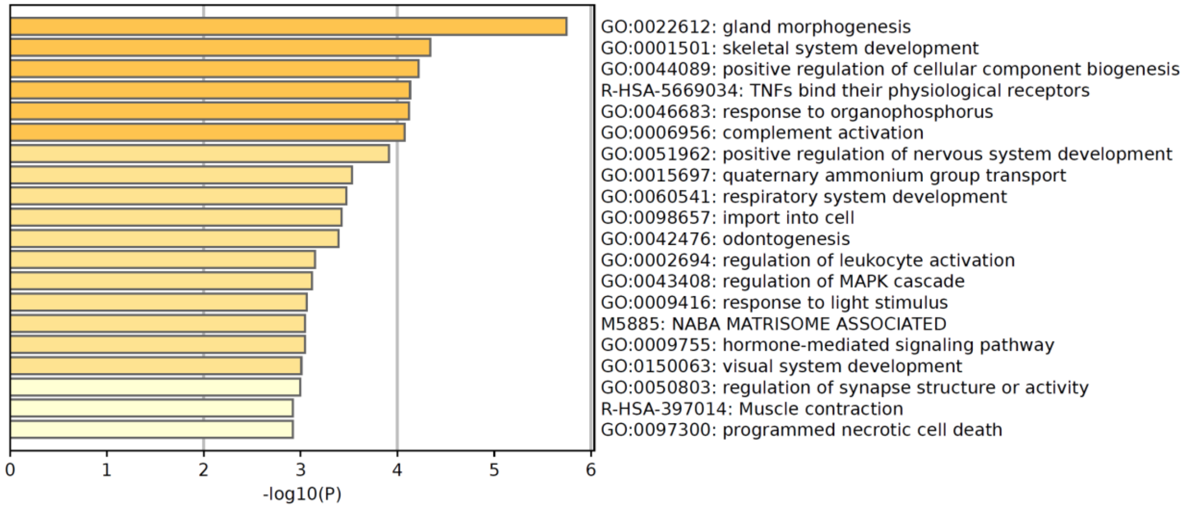

The activation of immune-related pathways in Cluster 1 in patients with long COVID.

Supplementary Figure 4

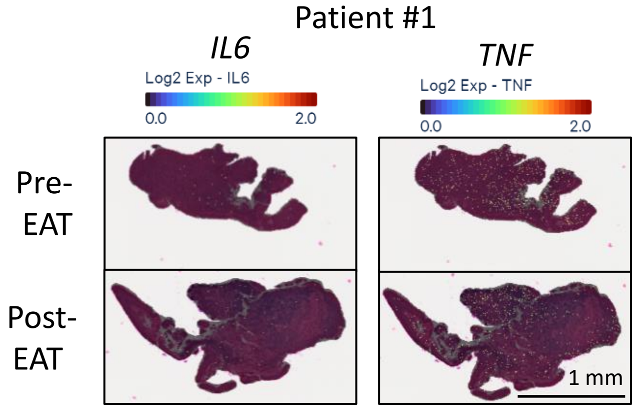

The spatial gene expression analysis of the inflammation-related cytokines interleukin-6 (IL-6) and tumour necrosis factor-α (TNF-α) in the epipharynx of Patient 1 before and after EAT treatment.

Supplementary Videos

Supplementary Video 1 demonstrates the endoscopic findings of the epipharynx observed at the initial visit prior to the application of epipharyngeal abrasive therapy (EAT) and during the EAT session for Patient 2. In contrast, Supplementary Video 2 presents the endoscopic findings of the epipharynx taken three months following the commencement of EAT, showcasing the post-EAT condition for the same patient.
